# Characterization of Four Orphan Receptors (GPR3, GPR6, GPR12 and GPR12L) in Chickens and Ducks and Regulation of *GPR12* Expression in Ovarian Granulosa Cells by Progesterone

**DOI:** 10.3390/genes12040489

**Published:** 2021-03-27

**Authors:** Zejiao Li, Biying Jiang, Baolong Cao, Zheng Zhang, Jiannan Zhang, Juan Li, Yan Huang, Yajun Wang

**Affiliations:** 1 Key Laboratory of Bio-Resources and Eco-Environment of Ministry of Education, College of Life Sciences, Sichuan University, Chengdu 610065, China; 15828227077@163.com (Z.L.); v_jby86@163.com (B.J.); cbl-scu@outlook.com (B.C.); zhang95424@outlook.com (Z.Z.); biozhangjn@scu.edu.cn (J.Z.); lijuanscuhk@163.com (J.L.); 2 China Conservation and Research Centre for the Giant Panda, Wolong Nature Reserve, Wolong 623006, China

**Keywords:** chickens, ducks, GPR3, GPR6, GPR12, GPR12L, ovary, progesterone

## Abstract

The three structurally related orphan G protein-coupled receptors, GRP3, GPR6, and GPR12, are reported to be constitutively active and likely involved in the regulation of many physiological/pathological processes, such as neuronal outgrowth and oocyte meiotic arrest in mammals. However, the information regarding these orphan receptors in nonmammalian vertebrates is extremely limited. Here, we reported the structure, constitutive activity, and tissue expression of these receptors in two representative avian models: chickens and ducks. The cloned duck *GPR3* and duck/chicken *GPR6* and *GPR12* are intron-less and encode receptors that show high amino acid (a.a.) sequence identities (66–88%) with their respective mammalian orthologs. Interestingly, a novel GPR12-like receptor (named GPR12L) sharing 66% a.a. identity to that in vertebrates was reported in the present study. Using dual-luciferase reporter assay and Western blot, we demonstrated that GPR3, GPR6, GPR12, and GPR12L are constitutively active and capable of stimulating the cAMP/PKA signaling pathway without ligand stimulation in birds (and zebrafish), indicating their conserved signaling property across vertebrates. RNA-seq data/qRT-PCR assays revealed that *GPR6* and *GPR12L* expression is mainly restricted to the chicken brain, while *GPR12* is highly expressed in chicken ovarian granulosa cells (GCs) and oocytes of 6 mm growing follicles and its expression in cultured GCs is upregulated by progesterone. Taken together, our data reveal the structure, function, and expression of GPR3, GPR6, GPR12, and GPR12L in birds, thus providing the first piece of evidence that *GPR12* expression is upregulated by gonadal steroid (i.e., progesterone) in vertebrates.

## 1. Introduction

G protein-coupled receptor 3 (GPR3), GPR6, and GPR12 are the three orphan receptors, which share about 60% amino acid (aa) sequence identity with each other [1]. Structurally, the three receptors are related to melanocortin receptors (MCRs), cannabinoid receptors (CBR), adenosine receptor (AR), sphingosine 1-phosphate (S1PR), and lysophosphatidic acid (LPAR) receptor [2,3]. To date, no endogenous ligands have been identified [4]. For these receptors, it was reported that sphingosine 1-phosphate (S1P) and dihydrosphingosine 1-phosphate (DHS1P) were ligands of GPR3, given that both could act on GPR3 to increase the intracellular cAMP level [5]. Paradoxically, several research teams could not reproduce this result [6,7]. Similarly, S1P was demonstrated to be a ligand of GPR6 [5], however, Yin et al. [6] failed to detect the agonistic activity of SIP on GPR6 [6]. Thus, the endogenous ligands for these receptors remain to be identified.

Although their endogenous ligands have not yet been identified, all three receptors are found to be constitutively active in mammals. It is reported that about 25% of the constitutively active GPCRs identified so far are found coupled to Gα_s_ and Gα_q_ proteins, and about 50% of these GPCRs couple to Gα_i/o_ proteins. In 1995, Eggerickx et al. [8] first discovered that GPR3 has constitutive activity and is coupled to Gs protein and can increase the intracellular cAMP level in the absence of ligands. Furthermore, they found that GPR3-induced cAMP level is equivalent to that of other ligand-activated Gs-coupled receptors. Subsequently, GPR6 and GPR12 also have been shown to constitutively activate the Gs-cAMP signaling pathway [5,9]. Together with the reports that many diseases are related to the constitutive activity of orphan GPCRs [10], increasing evidence showed that GPR3, GPR6, and GPR12 are likely coupled to the Gα_s_-cAMP/PKA signaling pathway and play important roles in the regulation of many physiological/pathological processes in mammals. For example, *GPR3* is reported to play a part in mammalian Alzheimer’s disease, obesity, and neuronal axonal growth [11,12,13,14]. *GPR6* is implicated in Parkinson’s disease, Alzheimer’s disease, and neuronal cell survival [14,15,16]. *GPR12* is reported to play vital roles in neurite outgrowth and neuronal development [14].

In addition to being related with the many central nervous system-related diseases, these GPRs are also reported to be involved in the meiosis arrest. In oocytes, a high cAMP level can continuously activate PKA, which phosphorylates and activates nuclear kinase Weel/MytI, which in turn inactivates cell division cycle 25B (CDC25B). Thus, as the activator of cyclin-dependent kinase 1 (CDK1), CDC25B can ultimately maintain the M-phase promoting factor (MPF) in an inactive state and prevent meiosis resumption [17,18]. In 2004, Mehlmann et al. [13] reported that GPR3 can maintain meiotic arrest in mouse oocytes through the Gα_s_ signaling pathway [13]. Later, in 2005, Mary Hinckley et al. [19] also found that rat oocytes only expressed GPR12, which is involved in the regulation of meiotic arrest [19], including preventing oocyte maturation and downregulation of oocyte *GPR12* expression to promotes meiotic resumption. In addition, in 2008, Deng et al. [20] found that overexpression of GPR3 in *Xenopus laevis* can increase cAMP levels to maintain meiotic arrest and the overexpression of GPR12 can prevent progesterone-induced meiosis resumption [20]. In 2012, Yang et al. [21] found that injection of specific small interfering double-stranded RNA (siRNA) complementary to GPR3 into pig oocytes can resume meiosis in early pre-antral follicles, in contrast, the overexpression of GPR3 by reinjecting of GPR3 mRNA can block this process again [21].

Although the constitutive activity of these orphan receptors seems to regulate the functions of the central nervous system (CNS) and oocyte meiotic arrest in mammals, our knowledge regarding the expression, function, and regulation of GPR3, GPR6, and GPR12 in nonmammalian vertebrate species is rather limited. Hence, using chickens and ducks as animal models, our present study aims to address (1) whether GPR3, GPR6, and GPR12 exist and function in birds; (2) whether these orphan receptors are expressed in the CNS and ovary. The results from this study will undoubtedly help to reveal the conserved roles of GPR3, GPR6, and GPR12 signaling across vertebrates.

## 2. Materials and Methods

### 2.1. Chemicals, Enzymes, Primers and Antibodies

All chemicals were purchased from Sigma-Aldrich (St. Louis, MO, USA). All primers used in this study were synthesized by Youkang Biotechnology Co., Ltd. (Chengdu, China) and are listed in Supplemental Table S1. The anti-CREB and anti-β-actin antibodies were purchased from Cell Signaling Technology Inc (CST, Beverly, MA, USA).

### 2.2. Animal Tissues

The adult chickens (1-year-old) or chicks (4-week-old) (Lohmann layer) were purchased from a local commercial company in Chengdu. Chickens were killed, and various tissues were collected. Granulosa cells (GCs) of 6 mm ovarian follicles from laying hens were collected for primary cell culture [22,23]. All animal experimental protocols used in this study were approved by the Animal Ethics Committee of College of Life Sciences, Sichuan University, and the assurance number is 2020030808 (8 March 2020).

### 2.3. RNA Extraction, RT-PCR, and Quantitative Real-Time PCR Assays

Total RNA was extracted from chicken tissues and cultured cells by RNAzol (Molecular Research Center, Cincinnati, OH, USA) according to the manufacturer’s instruction. Reverse transcription (RT) was performed by Moloney murine leukemia virus (MMLV) reverse transcriptase (Takara, Dalian, China). In brief, oligodeoxythymide (0.5 μg) and total RNA (2 μg) were mixed in a total volume of 5 μL, incubated at 70 °C for 10 min, and cooled at 4 °C for 2 min. Then, the first step buffer, 0.5 mM each of deoxynucleotide triphosphate and 100 U MMLV reverse transcriptase were added into the reaction mix, for a total volume of 10 μL. RT was performed at 42 °C for 90 min.

RT-PCR assay was performed to examine mRNA expression of *cGPR12* and *β-actin* genes in chicken 6–8 mm follicle oocytes, 6–8 mm follicle GCs, F5 follicle GCs, and F1 follicle GCs. PCR was performed under the following conditions: 2 min at 94 °C denaturation, followed by 39 cycles (30 sec at 98 °C, 30 s at 62 °C, and 15 s at 68 °C) of reaction, ending with a 20 min extension at 68 °C. The PCR products were visualized on a UV-transilluminator (Bio-Rad Laboratories, Inc. Hercules, CA, USA) after running electrophoresis on 2% agarose gel containing ethidium bromide.

According to a previously established method [24], quantitative real-time PCR (qRT-PCR) assay was performed to examine the mRNA expression of target genes in chicken tissues.

### 2.4. Cloning the cDNAs of Chicken, Duck, Zebrafish, and Pig GPR3, GPR6, GPR12, and GPR12L

According to predicted cDNA sequences of chicken *GPR*6 (XM_004940310.3), *GPR12* (XM_025146842.1), and *GPR12L* (XM_015278663.2) deposited in the GenBank, gene-specific primers were designed to amplify the 5′-cDNA and 3′-cDNA ends of *GPR6*, *GPR12*, and *GPR12L* from adult chicken brain. The amplified PCR products were cloned into pTA2 vector (TOYOBO, Osaka, Japan) and sequenced. Finally, the full-length cDNAs of *GPR6*, *GPR12*, and *GPR12L* were determined based on the sequences of 5′- and 3′-cDNA ends with overlapping regions.

Using RT-PCR, we also cloned the coding regions of *GPR3, GPR6, GPR12*, and *GPR12L* from duck, zebrafish, and pig brain tissues based on their predicted cDNA sequences deposited in the GenBank. Using genomic DNA extracted from human embryonic kidney 293 cells (HEK293) as the template, we also designed gene-specific primers and cloned the coding region of human *GPR3, GPR6*, and *GPR12* by PCR.

### 2.5. Sequence Alignment and Phylogenetic Analysis

We searched protein sequences of *GPR3*, *GPR6*, *GPR12,* and *GPR12L* genes in several vertebrates listed in Supplemental Table S2 (https://www.ncbi.nlm.nih.gov/). The deduced amino acid sequences were aligned using the ClustalW program (BioEdit, Carlsbad, CA, USA) [25]. The putative transmembrane (TM) domains were predicted by using an online protein topology prediction tool uniprot (https://www.uniprot.org/). To analyze the evolutionary relationship among vertebrate *GPR3*, *GPR6*, *GPR12,* and *GPR12L* genes (Supplemental Table S3), phylogenetic analysis was computed by using the program MEGA7 [26], in which the phylogenetic tree was constructed with maximum likelihood method, and confidence was estimated with 500 bootstrap replicates.

### 2.6. Detection of the Basal Constitutive Activity of Human, Pig, Chicken, Duck, and Zebrafish GPR3, GPR6, GPR12, and GPR12L

The expression plasmids encoding chicken (c) GPR6, cGPR12, and cGPR12L were prepared by cloning their complete open reading frames (ORFs) into the pcDNA3.1 (+) expression vector (Invitrogen, Waltham, MA). According to the cloned cDNA sequences of human *GPR3 (hGPR3), GPR6 (hGPR6),* and *GPR12 (hGPR12)*, pig *GPR3 (pGPR3), GPR6 (pGPR6),* and *GPR12 (pGPR12)*, duck *GPR3 (dGPR3), GPR6 (dGPR6), GPR12 (dGPR12),* and *GPR12L (dGPR12L)*, zebrafish *GPR3 (zfGPR3), GPR6 (zfGPR6), GPR12 (zfGPR12),* and *GPR12La (zfGPR12a)* deposited in the GenBank, the expression plasmids for these receptors were also prepared by cloning their ORFs into the pcDNA3.1 (+) expression vector (Invitrogen).

According to our previously established methods [27,28], all these receptors were transiently expressed in HEK293 cells and their basal constitutive activities were detected by dual-luciferase reporter assays. In brief, HEK293 cells were cultured in Dulbecco minimal Eagle medium (DMEM) supplemented with 10% (vol/vol) fetal bovine serum (Thermo Fisher Scientific Inc, Waltham, MA, USA), 100 U/mL penicillin G, and 100 μg/mL streptomycin (Life Technologies Inc., Grand Island, NY, USA) in a Corning Cell BIND 48-well plate (Corning, Tewksbury, MA, USA) and incubated at 37 °C with 5% CO_2_ for 24 h. A mixture containing 700 ng of pGL3-CRE-luciferase reporter construct, 700 ng of receptor expression plasmid or empty pcDNA3.1 (+) vector, 50 ng pL-TK vector, and 3 μL of jetPRIME (Polyplus-trans-fection SA, Illkirch, France) were prepared in 200 μL of jetPRIME buffer solution for 4 wells. Transfection was performed according to the manufacturer’s instruction when the cells reached 70% confluence. The cells were incubated for an additional 24 h at 37 °C before being harvested for dual-luciferase reporter assay. After the removal of culture medium, HEK293 cells were lysed by adding 100 μL of 1 × Cell Culture Lysis Buffer (Promega) per well, and the luciferase activity of 20 μL cellular lysates was determined with the luciferase assay kit (Promega, Madison, WI, USA). The luciferase activities in experimental groups were expressed as the relative fold increase compared to the control group transfected with empty pcDNA3.1(+) vector.

### 2.7. Western Blot

As described in our previous studies [29], HEK293 cells transfected with *cGPR6, cGPR12*, or *cGPR12L* expression plasmid were cultured on a 48-well plate at 37 °C for 24 h. Then, the cells were lysed and the phosphorylated CREB (pCREB), β-actin, phosphorylated ERK1/2 (pERK), and total ERK1/2 (tERK) were assayed by Western blot. pCREB levels were quantified using Image J program v1.8 (National Institutes of Health, Bethesda, MD, USA), normalized by that of intracellular β-actin, and then expressed as the relative fold increase compared to respective controls.

### 2.8. qPCR Detection of GPR12 Expression in Chicken Granulosa Cell (GC) and Oocytes

Total RNA was extracted from granulosa cells of 6–8 mm follicles, F1 follicles, and F5 follicles, according to our previously established method [30]. To extract the total RNA from oocytes of 6–8 mm follicles, four follicles were cut open by a pair of fine scissors to collect ooplasm (including the yolk) in a 2 mL tube pre-cooled by ice. The total RNA was then extracted from ooplasm by RNAzol. To examine the purity of total RNA extracted from the oocytes, the expression of bone morphogenetic protein 15 (*BMP15*) and growth differentiation factor 9 (*GFD9*) (GenBank accession no.: NC_006091.5 and NC_006100.5, respectively) in oocyte (and granulosa cells) was examined by qRT-PCR assay. As expected, *BMP15* and *GDF9* were detected in the oocyte nearly exclusively, but not in the granulosa cell layer, indicating the high purity of the total RNA extracted from oocytes.

### 2.9. Effect of E2, P4 and DHT5α on GPR12 Expression in Cultured 6–8 mm Follicle GCs

Granulosa cells (GCs) of 6–8 mm follicles were collected and digested by Collagenase 1 (Hyclone) at 37 °C for 20 min [30]. The dispersed GCs were cultured in Medium 199 supplemented with 15% fetal bovine serum in a Corning Cell BIND 48-well plate (Corning) at 37 °C with 5% CO_2_. After 4 h culture, the cells were treated with 0 nM, 1 nM, 10 nM, or 100 nM of gonadal steroids (E2, P4, DHT5α (dihydrotestosterone, an endogenous androgen sex steroid and hormone)) for 4 h and 24 h. Then, the total RNA was extracted with RNAzol reagent (Molecular Research Center) from cultured GCs and used for qPCR assay of *GPR12* mRNA levels. *β-actin* mRNA level was also examined as an internal control.

### 2.10. Promoter Analysis of Chicken GPR12 Gene

To determine whether the 5′-flanking region (near exon 1) of *cGPR12* displays promoter activities, we designed gene-specific primers and amplified their 5′-flanking regions (near exon 1) with high-fidelity KOD DNA polymerase (TOYOBO). The PCR products were cloned into pGL3-Basic vector (Promega) and sequenced. Finally, two promoter-luciferase reporter constructs of *cGPR12* (−3050/+277Luc and −1962/+277Luc) were prepared. In this experiment, the transcription start site (TSS) on exon 1 of *cGPR12* determined by 5′-RACE was designated as “+1”, and the first nucleotide upstream of TSS was designed as “−1”. Finally, the promoter activities of these constructs were examined in cultured chicken fibroblast cells line (DF-1) by the dual-luciferase reporter assay (Promega), as described in our previous study [31].

### 2.11. Detection of the Basal Constitutive Activity of GPR12 in Cultured GCs

To detect the basal constitutive activity of *GPR12* in cultured GCs, 100 ng of *cGPR12* expression plasmid, 100 ng of pGL3-CRE-luciferase reporter construct, and 10 ng pL-TK plasmid were co-transfected into GCs cultured in the Corning Cell BIND 48-well plate at a density of 5 × 10^4^ per well. After 24 h transfection, the luciferase activity was detected by dual-luciferase reporter assay, as described in our previous study [27,28].

### 2.12. Data Analysis

The mRNA levels of chicken *GPR12/GDF9/BMP15* were first calculated as the ratio to that of *β-actin* and then expressed as the fold difference compared to that of control/chosen group. The data was analyzed by one-way ANOVA followed by the Dunnett’s test in GraphPad Prism 7 (GraphPad Software, San Diego, CA, USA). To validate the results, all experiments were repeated at least twice.

## 3. Results

### 3.1. Cloning the Full-Length cDNAs of GRP3, GPR6, GPR12, and GPR12L in Chickens and Ducks

**Figure 1:**
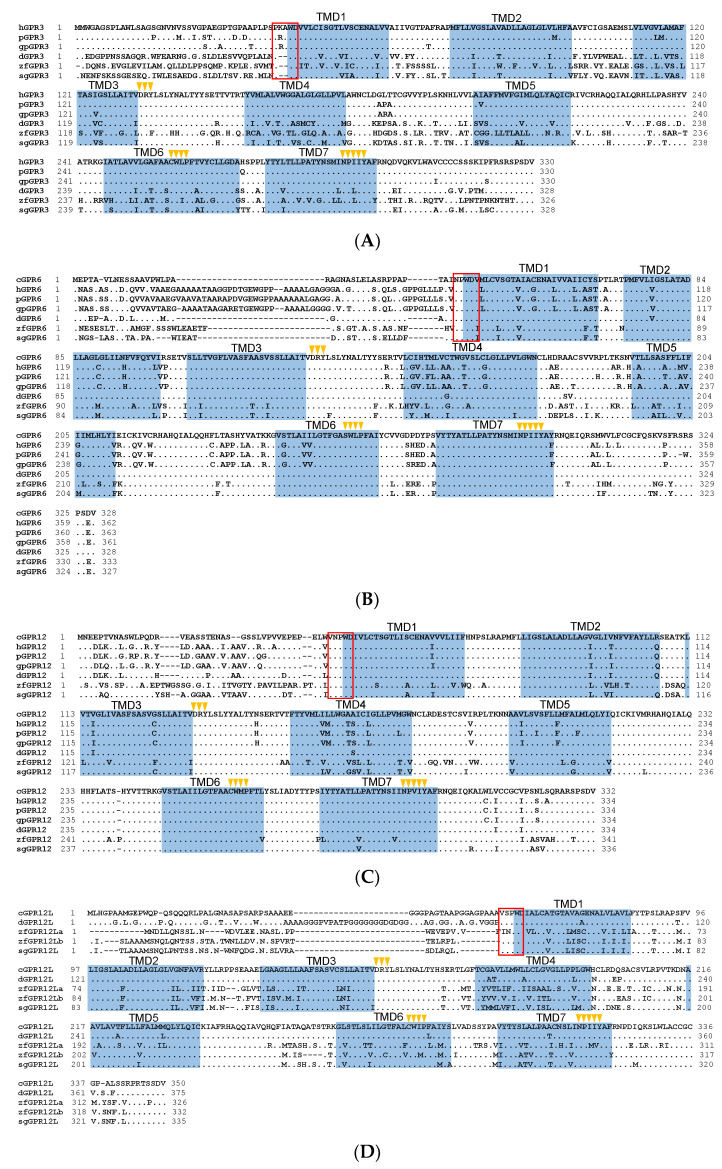

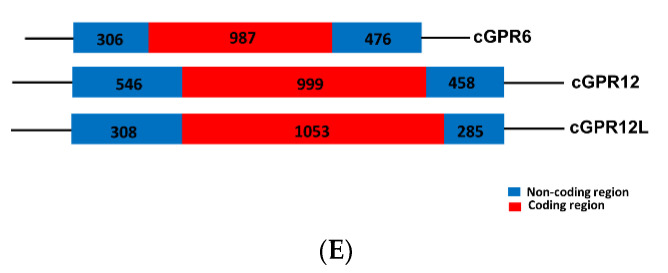
Amino acid sequence alignment of G protein-coupled receptor 3 (GPR3), GPR6, GPR12, and GPR12L in vertebrates. (**A**) Alignment of the cloned duck (d-)/zebrafish (zf-)/pig (p-) GPR3 with that of humans (hGPR3, NP_005272.1), giant pandas (gpGPR3, XP_034503372.1) and spotted gars (sgGPR3, XP_015204111.1); (**B**) Alignment of the cloned chicken(c-)/duck(d-)/zebrafish(zf-)/pig(p-) GPR6 with that of humans (hGPR6, NP_005275.1), giant pandas (gpGPR6, XP_002925693.2), and spotted gars (sgGPR6, XP_006626355.1); (**C**) Alignment of the cloned chicken (c-)/duck (d-)/zebrafish (zf-)/pig (p-) GPR12 with that of humans (hGPR12, NP_005279.1), giant pandas (gpGPR12, XP_002924260.1), and spotted gars (sgGPR12, XP_015196669.1); (**D**) Alignment of the cloned chicken (c-)/duck (d-)/zebrafish (zf-) GPR12L with that of anole lizard (lGPR12L, XP_028571577.1) and spotted gars (sgGPR12L, XP_015206591.1). The seven transmembrane domains (TMD1–7) and *N*-glycosylation sites (NXT/S) are shaded; the XXXWD motif is boxed; conserved motifs (DRY, XWXP, and NPXXY) are marked by arrowheads; (**E**) Exon–intron organization of chicken *GPR6*, *GPR12*, and *GPR12L*. The red boxes indicate the coding region, and the numbers inside denote the size (in bp); while the blue box represents the untranslated regions, (UTRs) and the number within specifies the size (in bp).

Using 5′- and 3′-RACE, we amplified and cloned the full-length cDNAs of *cGPR6* (Accession no. MW310573) and *cGPR12* (Accession no. MW310572) from adult chicken brain. In addition, we cloned a novel GPR12-like receptor from chicken brain and designated it as *GPR12L* (Accession no. MW310571). The coding regions of c*GPR6*, c*GPR12*, and c*GPR12L* genes are 987 bp, 999 bp, and 1053 bp long, respectively, which are predicted to encode receptors of 329, 333, and 351 amino acids (a.a.), respectively. Comparison of these cDNA sequences with the chicken genome database (GRCg6a, Ensembl release 101, http://www.ensembl.org/Gallus_gallus) revealed that c*GPR6*, c*GPR12*, and *cGPR12L* are intron-less (Figure 1E).

Using RT-PCR, we also cloned the coding region sequences of *GPR3*, *GPR6*, *GPR12*, and *GPR12L(s)* from brain tissues of duck (*dGPR3* (MW310577), *dGPR6* (MW310576), *dGPR12* (MW310575), and *dGPR12L* (MW310574)), zebrafish (*zfGPR3* (MW310585), *zfGPR6* (MW310584), *zfGPR12* (MW310583), *zfGPR12La* (MW310581), and *zfGPR12Lb* (MW310582)), and pig (*pGPR3* (MW310580), *pGPR6* (MW310579), and *pGPR12* (MW310578)).

Amino acid sequence alignment showed that 1) cGPR6 shows high a.a. sequence identity with GPR6 of ducks (96.3%), zebrafish (74.8%), pigs (67.0%), humans (67.1%), giant pandas (67.9%), and spotted gars (79.8%); 2) cGPR12 shows high a.a. sequence identity with GPR12 of ducks (94.9%), zebrafish (74.4%), pigs (87.1%), humans (88.3%), giant pandas (88.9%), and spotted gars (85.7%); 3) cGPR12L shows high a.a. sequence identity with GPR12L of ducks (84%), zebrafish (GPR12La, 54.5%; GPR12Lb, 61.60%), and spotted gars (63.6%) (Supplemental Table S4). Like human GPR6 and GPR12, chicken/duck/zebrafish GPR6, GPR12, and GPR12L have many conserved motifs and amino acid residues, characteristic of family A GPCR, such as a DRY motif for G protein coupling and two cysteine residues for a disulfide bond formation [32]. In addition, we noted a XXXWD motif near the first transmembrane domain, which is conserved across vertebrates (Figure 1A–D).

*GPR3* was cloned in ducks and zebrafish only, as this gene seems to be lost in chickens. Duck GPR3 is 329 a.a. long and shares high a.a. identity with GPR3 of humans (65.7%), pigs (66.3%), giant pandas (65.4%), zebrafish (49.6%), and spotted gars (74%) and a comparatively lower degree of sequence identity with chicken GPR6 (61.4%), GPR12 (62.1%), and GPR12L (48.2%) (Supplemental Table S4). Likewise, many structural features such as DRY, XWXP, and NPXXY motifs are present in duck GPR3 [32] (Figure 1A).

### 3.2. GPR3, GPR6, GPR12, and GPR12L in Birds and Other Vertebrates

To trace the evolutionary origin of chicken/duck *GPR3*, *GPR6*, *GPR12,* and *GPR12L*, we performed synteny analyses by searching the genes adjacent to *GPR3, GPR6, GPR12,* and *GPR12L* in chickens, ducks, humans, pigs, mice, turtles, zebrafish, and spotted gars.

**Figure 2:**
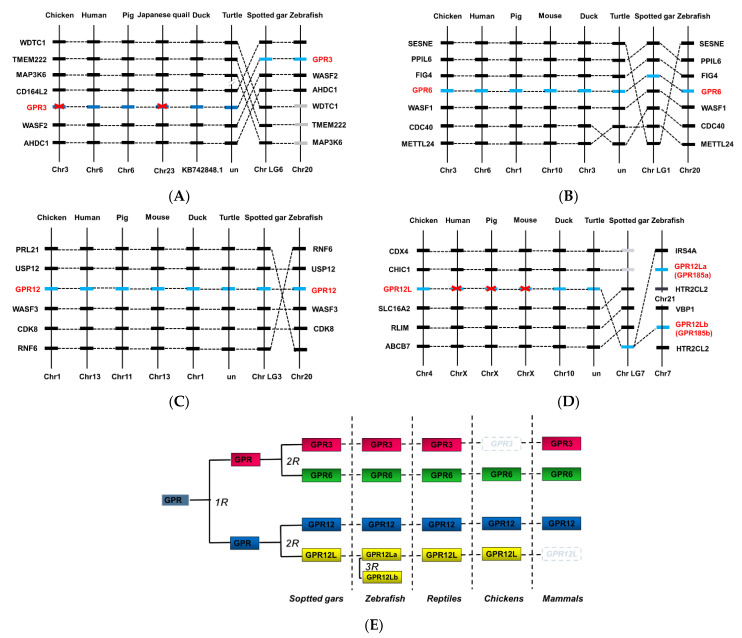
Synteny analysis of *GPR3*, *GPR6*, *GPR12*, and *GPR12L* in chickens and other vertebrates. *GPR3* (**A**), *GPR6* (**B**), *GPR12* (**C**), and GPR12L (**D**) are located in four syntenic regions conserved in vertebrate species including chickens, ducks, humans, pigs, turtles, spotted gars, and zebrafish. Dashed lines denote the genes of interest, while dotted lines indicate the syntenic genes identified in these species. Chr, chromosome. (**E**) Schematic diagram showing the existence of *GPR3*, *GPR6*, *GPR12,* and *GPR12L* in vertebrate species including spotted gars, teleosts (e.g., zebrafish), turtles, chicken, and mammals. *GPR3*, *GPR6*, *GPR12,* and *GPR12L* were likely duplicated from a common ancestral gene (denoted as “GPR”), which had undergone the two rounds of genome duplication event (2R) during early vertebrate evolution. The two *GPR12L* genes in zebrafish likely originated from the 3rd round (3R) genome duplication event occurred in teleost lineage.

As shown in Figure 2, *GPR6* and *GPR12* exist in all vertebrate species examined, indicating that chicken and duck *GPR6* and *GPR12* are orthologous to *GPR6* and *GPR12* in humans and other vertebrates. Duck *GPR3* is orthologous to *GPR3* of humans, zebrafish, and spotted gars, however, it is likely lost in chickens. *GPR12L*, which is a novel receptor identified in this study, exists in chickens, ducks, and other nonmammalian species, including zebrafish, however, it is likely lost in humans, pigs, and mice.

**Figure 3:**
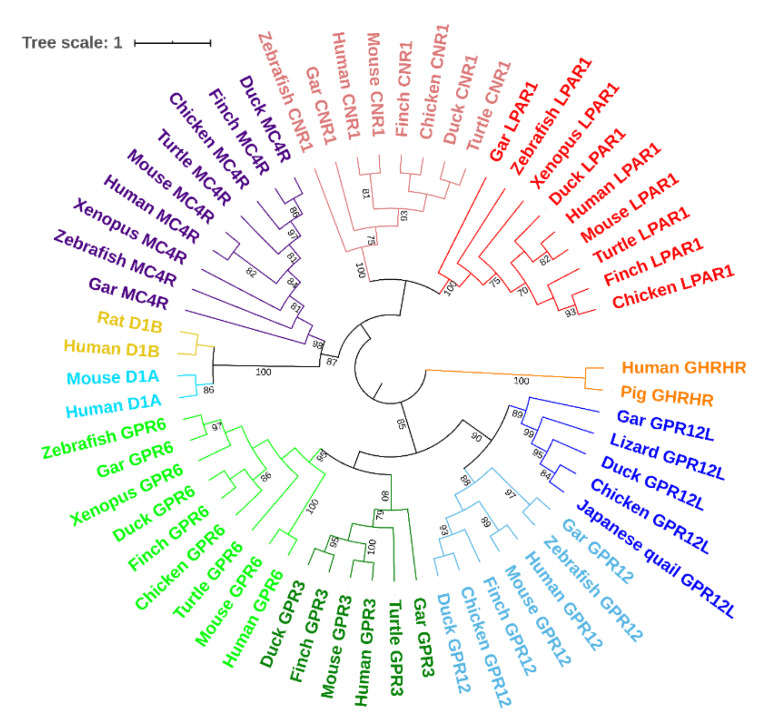
The phylogenetic tree of vertebrate GPR3, GPR6, GPR12, GPR12L, melanocortin 4 receptor (MC4R), cannabinoid receptor 1 (CNR1), and lysophosphatidic acid receptor 1 (LAPR1), dopamine receptor 1A (D1A), and dopamine receptors (D1B) constructed by MEGA7 software (maximum likelihood method). The GPR3 cluster is highlighted in deep green; the GPR6 cluster in orange; the GPR12 cluster in brown; the GPR12L cluster in purple.

To analyze the evolutionary relationship among vertebrate *GPR3*, *GPR6*, *GPR12,* and *GPR12L* genes, using the maximum likelihood method of MEGA7 software, we constructed a phylogenetic tree using the sequences deposited in the GenBank (Figure 3 and Supplemental Table S4). The results showed that *GPR3*, *GPR6*, *GPR12*, and *GPR12L* form a cluster, which is evolutionarily distant from another cluster formed by melanocortin 4 receptor (*MC4R*), cannabinoid receptor 1 (*CNR1*), and lysophosphatidic acid receptor 1 (*LPAR1*), and dopamine receptors 1A and 1B (*D1A* and *D1B*) of vertebrate species.

### 3.3. Detection of the Constitutive Activity of GPR3, GPR6, GPR12 and GPR12L in Birds

**Figure 4:**
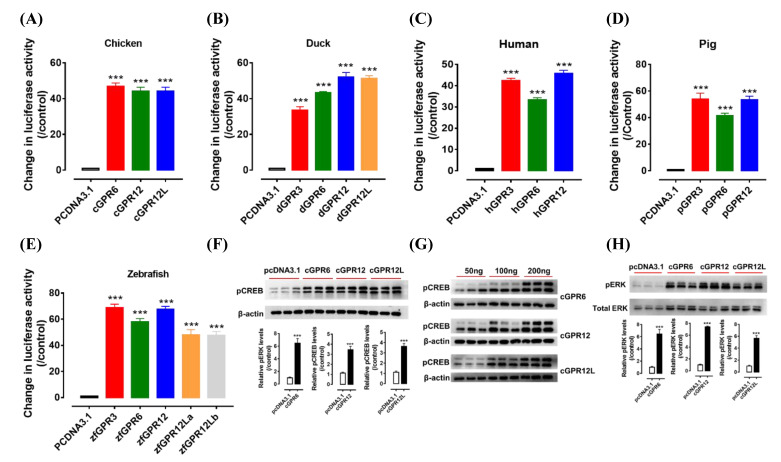
Constitutive activity of GPR3, GPR6, GPR12, and GPR12L(s) in chicken (**A**), duck (**B**), human (**C**), pig (**D**), and zebrafish (**E**). The activity of each receptor expressed in human embryonic kidney 293 (HEK293) cells was detected by dual-luciferase reporter assay. Each data point represents the mean ± SEM of 4 replicates (*N* = 4). ***, *p* < 0.001 vs. control (cells transfected with empty pcDNA3.1(+) vector). Western blot detection of the phosphorylated CREB (pCREB, (**F**)) and ERK (pERK, (**H**)) in HEK293 cells expressing cGPR6, cGPR12, and cGPR12L. pCREB and pERK levels were first normalized by that of actin and total ERK (tERK), respectively, and then expressed as the fold increase compared with the control group (transfected with empty pcDNA3.1(+) vector). ***, *p* < 0.001 vs. control. (**G**) The levels of pCREB increase dose-dependently in HEK293 cells transfected with increasing amount of cGPR6, cGPR12, or cGPR12L expression plasmid (50 ng, 100 ng, and 200 ng). Note. zebrafish has two *GPR12L* genes (*GPR12La* and *GPR12Lb*).

It is reported that mammalian GPR3, GPR6, and GPR12 have basal constitutive activity and can activate the Gs-cAMP signaling pathway [5,8,9]. To determine whether avian GPR3, GPR6, GPR12, and GPR12L have basal constitutive activity, we detected the constitutive activity of these receptors expressed in HEK293 cells using dual-luciferase reporter assays. As shown in Figure 4A–D, like human and pig GPR3, GPR6, and GPR12, chicken GPR6, GPR12, and GPR12L and duck GPR3, GPR6, GPR12, and GPRL12L have high basal constitutive activity and the luciferase activity of HEK293 cells expressing these receptors is more than 30-fold higher than that of the control group. Similarly, five zebrafish receptors (zfGPR3, zfGPR6, zfGPR12, zfGPR12La, and zfGPR12Lb) also display strong constitutive activity under the same condition (Figure 4E). All these findings indicate that GPR3, GPR6, GPR12, and the novel GPR12L are constitutively active in vertebrates including birds.

Using Western blot, we found that transfection of chicken GPR6, GPR12, and GPR12L into HEK293 cells for 24 h can dose-dependently enhance phosphorylation level of CREB (pCREB), a downstream mediator of the cAMP/PKA signaling pathway (Figure 4F,G). All these findings support the hypothesis that all the orphan receptors identified in chickens can activate the Gα_s_-cAMP/PKA/CREB signaling pathway.

Interestingly, we found that chicken GPR6, GPR12, and GPR12L expressed in HEK293 cell can enhance ERK phosphorylation (Figure 4H) [33].

### 3.4. Tissue Expression of GPR3, GPR6, GPR12, and GPR12L in Chickens and Ducks

**Figure 5:**
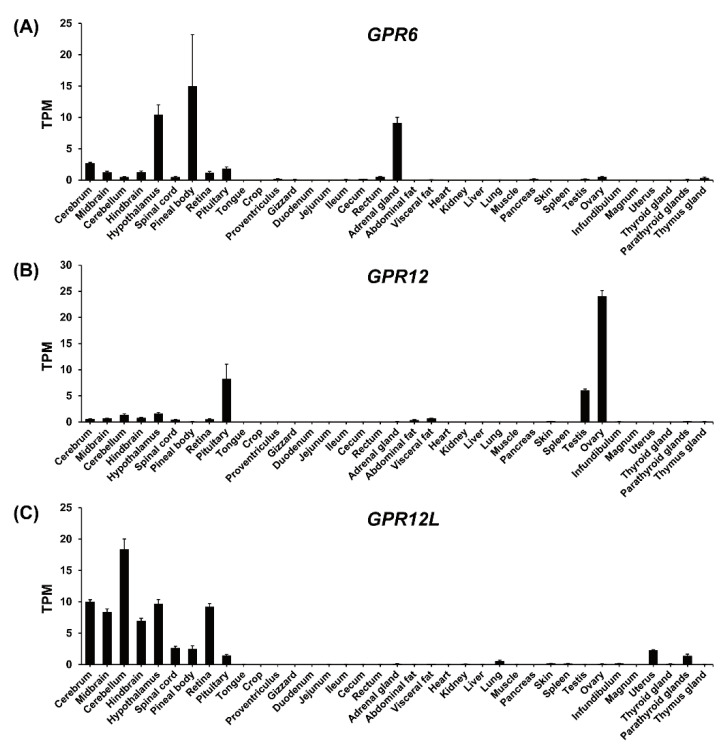
RNA-seq data analysis showing the expression of *GPR6* (**A**), *GPR12* (**B**), and *GPR12L* (**C**) in adult chicken tissues. Each data point represents the mean ± SEM of 6 individual adult chickens (3 males and 3 females) (*N* = 6).

To examine the tissue distribution of *GPR6*, *GPR12,* and *GPR12L* in adult chickens, we analyzed the expression of the three receptors in 37 chicken tissues with reference to the RNA-seq data previously obtained in our lab. We found that *GPR6* and *GPR12L* are widely expressed in the central nervous system (CNS). *cGPR6* shows a high mRNA level in the hypothalamus, cerebrum, and pineal body, while c*GPR12L* has a high mRNA level in the hypothalamus, cerebrum, cerebellum, hindbrain, midbrain, and retina. In addition, *GPR6* and *GPR12L* are also expressed in several peripheral tissues, including the adrenal gland (*GPR6*), pituitary (*GPR6*, *GPR12L*), uterus (*GPR12L*), and parathyroid gland (*GPR12L*). Weak or nondetectable expression of *GPR6* and *GPR12L* was found in other tissues examined. In contrast, c*GPR12* is predominantly expressed in the ovary, testes, and anterior pituitary (Figure 5).

### 3.5. Regulation of GPR12 Expression in Ovarian Granulosa Cell (GC) by Steroid Hormones

**Figure 6:**
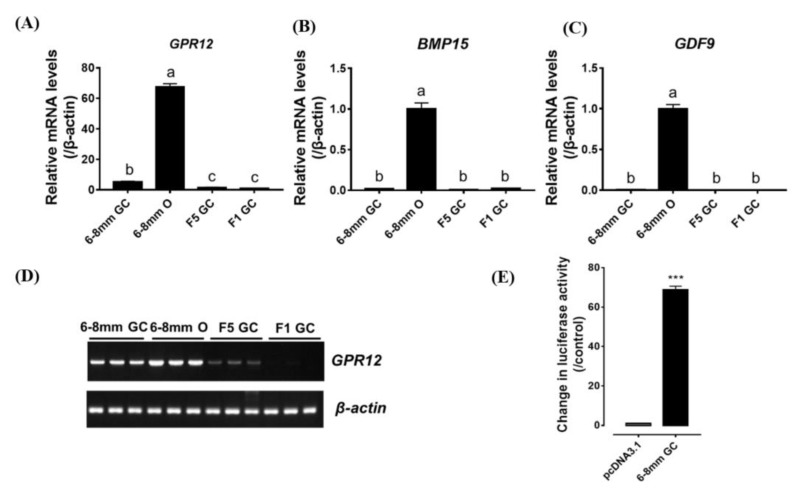
(**A**) qPCR of GPR12 mRNA expression in oocytes (6–8 mm Oo) of chicken 6–8 mm follicles and granulosa cells of 6–8 mm (6–8 mm GC), F5 (F5-GC) and F1 follicles (F1-GC). (**B**,**C**) Predominant expression of BMP15 (**B**) and GDF9 (**C**) mRNA in 6–8 mm GC, 6–8 mm Oo, F5-GC and F1-GC. In graphs A–C, different letter indicates the statical difference between two groups. (**D**) RT-PCR detection of GPR12 mRNA expressed in chicken 6–8 mm GC, 6–8 mm Oo, F5-GC, and F1-GC. The GPR12 band intensity is the strongest in 6–8 mm Oo, and its signal in GC cells decreases along follicle development. No PCR band was detected in all reverse transcription (RT)-negative controls. In graphs A–D, each data point represents the mean ± SEM of 4 replicates (*N* = 4). (**E**) The constitutive activity of GPR12 in cultured 6 mm GC transfected with cGPR12 plasmid as detected by dual-luciferase reporter assay. Each data point represents the mean ± SEM of four replicates (*N* = 4). *** *p* < 0.001 vs. control.

Since only *GPR12* is predominantly expressed in chicken ovary, we further examined its expression in the GC layer of developing ovarian follicles, including 6–8 mm prehierarchy follicles, F5 and F1 preovulatory follicles by qRT-PCR assay. As shown in Figure 6A, c*GPR12* expression in the GC is stage-dependent. It is highly expressed in the GC of 6 mm follicles, and its expression decreases gradually in the GCs of F5 and F1 follicles.

Interestingly, using qRT-PCR or RT-PCR assay, we found that *GPR12* has a comparatively high expression level in the oocytes (of 6 mm follicles) (Figure 6B–D), where there is previous report of abundant expression of *BMP15* and *GDF9* [30].

**Figure 7:**
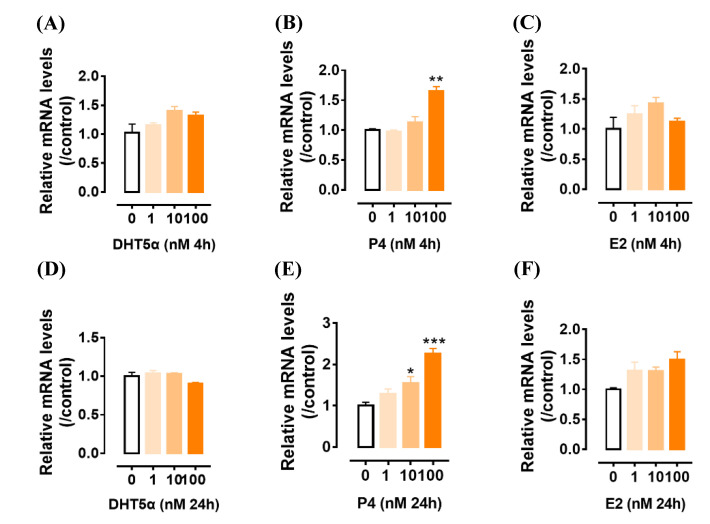
The effects of androgen (DHT5α) (**A**,**D**), progesterone (P4) (**B**,**E**), and estradiol (E2) (**C**,**F**) treatment (0 nM, 1 nM, 10 nM, and 100 nM) for 4 h and 24 h on c*GPR12* mRNA expression in cultured granulosa cells from 6–8 mm follicles as detected by qPCR. Each data point represents the mean ± SEM of 4 replicates (*N* = 4). * *p* < 0.05; ** *p* < 0.01; *** *p* < 0.001 vs. control.

The expression of *GPR12* in gonads led us to speculate that its expression in the GCs may be regulated by gonadal steroids, such as estradiol (E2), androgen (DHT5α), and progesterone (P4). To test this, the GCs collected from 6 mm follicles were cultured and treated by E2, DHT5α, and P4, and *GPR12* mRNA levels were examined by qRT-PCR. As shown in Figure 7, P4 treatment for 4 h or 24 h can increase *GPR12* expression dose-dependently, while E2 and DHT5α seem capable to slightly increase *GPR12* expression.

### 3.6. Identification of the Promoter Regions of Chicken GPR12

**Figure 8:**
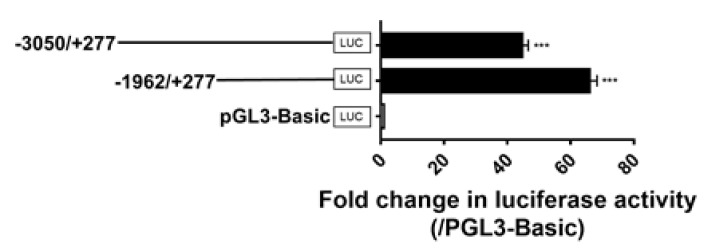
Detection of promoter activities of the 5′-flanking region of chicken *GPR12* in cultured chicken fibroblast cells line (DF-1). Various lengths of the 5′-flanking regions of *cGPR12* were cloned into pGL3-Basic vector for the generation of multiple promoter-luciferase constructs (−3050/+277 Luc and −1962/+277 Luc). These promoter-luciferase constructs were then co-transfected into DF-1 cells along with pRL-TK vector, and their promoter activities were determined by dual-luciferase reporter assays. The transcriptional start site identified by 5′-RACE was designated as “+1” (see Figure S1). Each value represents the mean ± SEM of four replicates (*N* = 4). *** *p* < 0.001 vs. pGL3-Basic vector.

Since the 5′-untranslated regions (5′-UTR) of *cGPR12* was determined by 5′-RACE PCR (Figure 1D), it led us to speculate that the promoter region(s) driving *GPR12* transcription may be located upstream of the 5′-UTR. To test this notion, we cloned the 5′-flanking regions of *cGPR12* into the pGL3-Basic vector and tested their promoter activities in cultured DF-1 cells. As shown in Figure 8, the 5′-flanking regions (from −3050 to +277) of *cGPR12* display promoter activity in DF-1 cells.

Using a promoter deletion approach, we noted that the region from −1962 to +277 is capable to display strong promoter activities, hinting that the core promoter region of c*GPR12* is likely located within this region. Using the AnimalTFDB 3.0 database [34], filtered with Q values less than 0.01, binding sites for many transcription factors such as Sp1, USF, E2F, and AP2 were predicted to exist within this promoter region (−1962/+277) (Figure S1). However, whether they are functional cis-regulatory elements requires further investigation.

## 4. Discussion

In this study, the coding regions of chicken and duck (and zebrafish) *GPR3*, *GPR6*, *GPR12,* and *GPR12L* were cloned. Functional assays demonstrated that avian GPR3, GPR6, GPR12, and GPR12L can constitutively activate the Gs-cAMP/PKA signaling pathway. RNA-Seq and qPCR assays indicated that *GPR6* and *GPR12L* are highly expressed in the CNS, revealing their active involvement of CNS function. In the present study, *GPR12* was found to be highly expressed in ovarian GCs and oocytes of 6 mm growing follicles in chickens. In combination with the observance that progesterone can upregulate *GPR12* expression in the GCs, thus implying the active role of *GPR12* in meiotic events. To our knowledge, our study represents the first to report the expression and functionality of the four orphan receptors in birds.

### 4.1. Identification of GPR3, GPR6, GPR12, and GPR12L in Birds

Although *GPR3, GPR6,* and *GPR12* are predicted to exist in nonmammalian vertebrates, their structure, expression, and functionality have not been studied. The present study for the first time reported the cDNAs of *GPR3, GPR6, GPR12,* and the novel *GPR12L* from chicken, ducks, and zebrafish, confirming that these orphan receptors are expressed in nonmammalian vertebrate species, including birds. Interestingly, we can only identify *GPR6*, *GPR12,* and *GPR12L* from chicken brain, while *GPR3* is likely lost in chickens, as revealed by synteny analysis. Considering the relatively low degree of a.a. sequence identity of GPR3 among vertebrates (Supplemental Table S4) and the functional redundance of these orphan receptors, it is not surprising that *GPR3* might have been lost during speciation, resulting in its absence in some avian species, e.g., in chickens, quails, and turkeys. In the present study, sequence analyses revealed that all these receptors share common conserved motifs and structural features. For example, they all retain DRY, XWXP, and NPXXY motifs present in TM3, TM6, and TM7, respectively [32]. In this study, we also found that GPR12L, the novel receptor identified in chickens and ducks, also shares these conserved motifs. Despite the high structural similarity shared among these receptors (Supplemental Table S4), the differences in their N- and C-termini, extracellular loops, intracellular loops, and transmembrane regions were observed.

In the present study, the sequence analyses indicated a relatively low a.a. sequence identity (Supplemental Table S4, 60% a.a. identity) among four structurally related orphan receptors, including the novel *GPR12L*. Although low sequence identity exists between orthologous GPR receptors, all these genes could be classified into a homologous subfamily, reference to research in olfactory receptors [35]. Together with the phylogenetic tree, our study showing the closer evolutionary relationship among *GPR3*, *GPR6*, *GPR12,* and *GPR12L* led us to propose that the four receptors are likely originated from a common ancestral *GPR* gene, which had experienced two rounds of genome duplication (2R) during early vertebrate evolution, thus resulting in the current receptor repertoire in higher vertebrate species such as spotted gars, turtles, and birds. In addition, the third round of genome duplication (3R) in the teleost lineage likely resulted in the presence of two *GPR12L* genes (*GPR12La*, *GPR12Lb*) in some teleosts (e.g., zebrafish) (Figure 2E).

In this study, regardless of the absence or presence of their ligands, all the four receptors from chickens, ducks, zebrafish, pigs, and humans are proven to be constitutively active. As shown in Figure 4, without stimulation by any ligands, the expression of GPR3, GPR6, GPR12, and GPR12L caused more than 30-fold increase in luciferase activities of HEK293 cells when compared with the control group. The present study is in accordance with the reports from mouse and rat [13,16,19], supporting their active roles across species. Interestingly, we noted that chicken GPR6, GPR12, and GPR12L expression can also enhance ERK phosphorylation levels. Similar signal pathways have been detected in mouse, showing that GPR3 and GPR6 are able to mediate the anti-apoptotic effect of PC cells through extracellular signal-regulated kinase 1/2 (ERK1/2) [36,37]. In addition, overexpression of GPR12 may induce PC12 cells to differentiate into neuron-like cells by activating the ERK1/2 signaling pathway [38]. The constitutive activities shared by these receptors, including GPR3, GPR6, GPR12, and GPR12L, suggest that these receptors may exert important roles in target tissues, similarly to their mammalian counterparts [39].

### 4.2. Tissue Expression of GPR3, GPR6, GPR12, and GPR12L in Chickens and Ducks

Understanding the tissue distribution of genes is important to probe their physiological roles in a species. In the present study, *GPR6* was detected to be highly expressed in the adrenal gland and CNS, including the hypothalamus in chickens. Our findings are in part consistent with those findings in mammals where *GPR6* is mainly located in the striatum and hypothalamus and participates in neurite outgrowth, thus being related with Alzheimer’s disease, Parkinson’s disease, and instrumental learning [14,15,16,40,41]. In the present study, the expression of *GPR6* was not detected in stomach and testes, in contrast with the observance from Morales et al. [1].

In the present study, the expression of *GPR12* was found to be expressed in a wide range of tissues, including pituitary, testis, ovary, and the brain subregions. Of the multiple tissues, the *GPR12* was found to be expressed abundantly in pituitary, testis, and ovary. The tissue distribution of *GPR12* in chicken was partially in accordant with that in human, where *GPR12* were detected in the eyes, breast, liver, and skin and the brain subregions, including cerebral cortex, striatum, pituitary, and cerebellum [42,43]. It was reported that *GPR12* expression is associated with neurite outgrowth and neuronal development, cell survival, proliferation, and carcinogenesis [14,19]. In this study, using qRT-PCR, we found that c*GPR12* is highly expressed in oocytes and GCs of 6 mm prehierarchy follicles and its expression decreases gradually towards the follicle maturation and is the lowest in F1 follicles. Our data are similar to the finding in rodents, in which *GPR12* and *GPR3* are expressed in oocytes [19].

In the present study, the novel gene *GPR12L* was detected to be highly expressed in brain subregions including cerebrum, midbrain, cerebellum, hindbrain, hypothalamus, spinal cord, pineal body, retina, and pituitary. In combination with the observance that GPR3 is lost in chicken and there is a low abundance of GPR12 in brain subregions, the novel gene GPR12L may partially replace the function of GPR3 in the system.

Similarly to the expression profiles of GRP6, GPR12, and GPR12L detected in chickens, *GPR6* are highly expressed in the brain, while *GPR12* and *GPR12L* are highly expressed in the gonads and brain in ducks (Figure S2C). Since GPR3 is found to exist in ducks, tissue expression analysis revealed that GPR3 is highly expressed in tissue brain and gonads, similarly to the study by Tanaka et al., [11,12,13,14,44,45].

In spotted gars and zebrafish, we also noted that *GPR3*, *GPR6*, *GPR12,* and *GPR12L* are highly expressed in the brains and gonads (Figure S2). The conserved expression of these receptors in the brain and gonads among vertebrates strongly suggests that the four “orphan” receptors play crucial roles in these tissues.

### 4.3. GPR12 is Expressed in Oocytes and GCs of 6-mm Growing Follicles

In the present study, the high abundance of c*GPR12* is found to be expressed in oocytes and the GCs of 6 mm prehierarchy follicles and its expression decreases gradually towards the follicle maturation. Together with the observance that *GPR3* is likely lost in the chicken genome, the predominant expression of cGPR12 in oocytes and the surrounding GCs of chicken growing follicles (6 mm), in combination with its mediation of cAMP/PKA signaling pathway (Figure 6E), strongly supports its involvement in oocyte meiosis arrest. In vertebrates, oocyte meiosis will arrest at the diplotene stage for a long time. Then, oocyte meiosis will resume at the stage of sexual maturity when pituitary luteinizing hormone (LH) surges to trigger the events. The high cAMP level within the oocytes is reported to be necessary to maintain the meiosis arrest [46]. It is reported that the high cAMP concentration may be from the cumulus cells that enter into the oocyte through gap junctions [47]. In the later study, a report showed that the cAMP may be produced by the oocytes themselves through the Gα_s_ signaling under hormone stimulation [48]. In 2004, Kalinowski et al. [49] found that the injection of negative-domain form of Gα_s_ into oocytes of mice, *Xenopus,* and zebrafish can lead to the resumption of meiosis in oocytes [49].

The abundant expression of *GPR12* in GCs led us to examine whether gonadotropin and gonadal steroids can regulate *GPR12* expression in GCs. Our study showed that FSH or LH cannot regulate *GPR12* expression in vitro. However, we found that P4 can significantly increase *GPR12* expression in cultured GCs from 6–8 mm follicles in a time- and dose-dependent manner, while E2 and DHT5α only increase *GPR12* expression slightly. Considering that P4 is predominantly expressed in GCs of the largest preovulatory F1 follicles, the upregulation of *GPR12* induced by P4 in these follicles may enhance cAMP levels, which will further maintain the high cAMP levels within the oocytes through gap junction [50,51]. Our data provide the first piece of evidence that gonadal steroid, i.e., P4, can upregulate *GPR12* expression in the gonads of vertebrates. The identification of the core promoter region of chicken *GPR12* is also helping to explore the detailed regulation mechanism by P4 in further investigation.

## 5. Conclusions

In summary, four orphan receptors, namely, GPR3, GPR6, and GPR12, and a novel GPR12-like receptor (GPR12L), were cloned in chickens/ducks and other vertebrate species in this study. Functional study elucidated that GPR3, GPR6, GPR12, and GPR12L are constitutively active and capable of increasing intracellular cAMP levels. RNA-seq assays revealed that avian *GPR3*, *GPR6,* and *GPR12L* are mainly expressed in the brain and *GPR12* is highly expressed in the pituitary and gonads. Moreover, we observed that *GPR12* is highly expressed in the oocytes and GCs of growing follicles and its expression in GC is likely regulated by P4. Our data provide valuable insights into the function, tissue expression, and roles of these orphan receptors in vertebrates under a comparative endocrinology perspective.

## Data Availability

Data sharing not applicable.

## Supplementary Materials

The following are available online at https://www.mdpi.com/article/10.3390/genes12040489/s1, Figure S1: Analysis of the promoter region of cGPR12, Figure S2: RNA-seq data analysis showing the tissue expression of GPR3, GPR6, GPR12, GPR12La (GPR185a), and GPR12Lb (GPR185b) in spotted gars (A) and zebrafish (B) duck (C) tissues, Table S1: Primers used in this study, Table S2. Lists of the GPCR sequences, their GenBank accession numbers and species used in amino acid sequence alignment, Table S3. Lists of genes and their GenBank accession numbers used to generate the phylogenetic tree in this study, Table S4. Amino acid sequence identity of GPR3, GPR6, GPR12, GPR12L among vertebrate species including chickens, humans, ducks, zebrafish, and spotted gars.
