# Supplementary material for: Characterization of Four Orphan Receptors (GPR3, GPR6, GPR12 and GPR12L) in Chickens and Ducks and Regulation of GPR12 Expression in Ovarian Granulosa Cells by Progesterone
Source: Genes (Basel). 2021 Mar 27;12(4):489. doi: 10.3390/genes12040489 (PMC8065388; doi:10.3390/genes12040489)
Supplement: Supplementary file 1 [file genes-12-00489-s001.pdf]

Characterization of Four orphan receptors (GPR3, GPR6, GPR12 and GPR12L) in Chickens and Ducks and Regulation of *GPR12* Expression in Ovarian Granulosa Cells by Progesterone

Zejiao Li[1], Biying Jiang[1], Baolong Cao[1], Zheng Zhang[1], Jiannan Zhang[1], Juan Li[1], Yan Huang[2]* and Yajun Wang[1]*

Supplementary materials

Table S1. Primers used in this study

Gene	Primer name	Primer sequences (5’-3’)
Primers for cloning the 5’UTR		
cGPR6	raL1	ATGAGGCCGAGCCCGGCCAGCAGGTC
	raL2	ACACACAGCATCACGTCCCAGGGGTT
cGPR12	raL1	AGTGATGGCCAGCAAGCTGCCAACGG
	raL2	CCTATGAGCAGGAACATGGGCGCGCG
cGPR12L	raL1	CAGCAGGTCGGCCAGCGCCAGGCTGC
	raL2	CCCGTGGCGCACAGAGCGATGTCCC
Primer for cloning the 3’-UTR		
cGPR6	raU1	GCAGACATGCCCATCAGATAGCTCTC
	raU2	TTACGCCACGCTTCTACCTGCTACCT
cGPR12	raU1	CGTGATGCGCCATGCCCATCAGATTGC
	raU2	GCTGCTTGCTGGATGCCTTTCACGCT
cGPR12L	raU1	CCTCCACCCGCAAAGGGCTCTCCACG
	raU2	CGCCGTGTACACGTACTCCCTGGCGC
[a]*Primer for cloning the coding regions*		
cGPR6	U1	AGTCCAGTGTGGTGgaattcGCGGCGATGGAGCCAACGGC
cGPR6	L1	TGGATATCTGCAgaattcTCAGACATCACTAGGGGA
cGPR12	U1	AGTCCAGTGTGGTGgaattcGTTAAAATGAATGAAGAGCC
cGPR12	L1	GCTGGATATCTGCAgaattcTCAGACATCGCTGGGCGATC
cGPR12L	U1	AGTCCAGTGTGGTGgaattcCTTTCGATGCTGCACGGCCC
cGPR12L	L1	GCTGGATATCTGCAgaattcTCACACGTCGCTGGATGTCC
dGPR3	U1	CCAGTGTGGTGgaattcGGCAGCATGATGGAGGACG
dGPR3	L1	TGCAgaattcGTCACTCAGACGTCGCTGGGCGGACCT
dGPR6	U1	CAGTGTGGTGgaattcGAGGAGATGGAGCCAGAGCC
dGPR6	L1	ATCTGCgaattcGTCACTCAGACATCGCTAGGGGA
dGPR12	U1	TCCAGTGTGGTGgaattcGTTAAAATGAATGAAGATCT
dGPR12	L1	TCTGCAgaattcGTCACTCAGACATCACTGGGCGAT
dGPR12L	U1	CAGTGTGGTGgaattcCCAGCGATGCTGCACGGCCCC
dGPR12L	L1	TATCTGCAgaattcGTCACTCACACGTCGCTGGACGT
hGPR3	U1	AGTCCAGTGTGGTGgaattcGGTACCATGATGTGGGGTGC
hGPR3	L1	ATATCTGCAgaattcGTCACCTAGACCATCACTGGGGGAGC
hGPR6	U1	CAGTGTGGTGgaattcGCCGCGATGAACGCGAGCGC
hGPR6	L1	ATCTGCAgaattcGTCACTCAGACCTCACTGGGGGA
hGPR12	U1	AGTCCAGTGTGGTGgaattcGTTAAAATGAATGAAGACCT
hGPR12	L1	ATATCTGCAgaattcGTCACCTACACATCACTGGGCGAGC
pGPR3	U1	GgaattcCGGTACCATGATGTGGGGTG
pGPR3	L1	GgaattcCTAGACGTCACTGGGAGAA

pGPR6	U1	GgaattcCGCCGCGATGAACGCGAGCG
pGPR6	L1	GgaattcCTCAGACCTCACTGGGGGACC
pGPR12	U1	GgaattcCATGAATGAAGACCTGAAGGTC
pGPR12	L1	GgaattcCCTACACATCACTGGGCGAC
zGPR3	U1	AGTCCAGTGTGGTGgaattcTTCCTAATGGACCAGAACAG
zGPR3	L1	ATATCTGCAgaattcGTCACTCAGACGTCGCTGGGGGTGT
zGPR6	U1	AGTCCAGTGTGGTGgaattcTCAGCGATGAACGAGAGCGA
zGPR6	L1	ATATCTGCAgaattcGTCACCTAGACCTCACTGGGCGATC
zGPR12	U1	AGTCCAGTGTGGTGgaattcGGCAGAATGAGTGAAGAGGT
zGPR12	L1	ATATCTGCAgaattcGTCACTCAGACGTCGCTGGGGGTCC
zGPR12La	U1	TCCAGTGTGGTGgaattcTGAGCTATGATTCACTCTCT
zGPR12La	L1	TGGATATCTGCAgaattcCTATACATCGCTCGATGT
zGPR12Lb	U1	TCCAGTGTGGTGgaattcTGAGCTATGATTCACTCTCT
zGPR12Lb	L1	TGGATATCTGCAgaattcCTATACATCGCTCGATGT
Primers for RT-PCR and quantitative RT-PCR assays		
cGPR12	U1	CAGGACCGCGTAGAAGCCAG
cGPR12	L1	TGGCTTCGGAGCGCAGCAG
BMP15	U1	GGCCGCAGTCTGAGCACCAA
BMP15	L1	GGCAGCAGGCAGCAGCTCCA
GDF9	U1	CCGTGTTCTGAGTGCCAGCAC
GDF9	L1	CACAGACAAATGGCACGTGC
Primers for amplifying cGPR12 promoter regions		
cGPR12 (-3050/+277)	U1	ATCTGCGATCTAAGTAAGCTCCACCAAGGTACAAGC
cGPR12 （-1962/+277）	U1	ATCTGCGATCTAAGTAAGCTTCCTCTGACAGAGGATG
cGPR12	L1	AAGCTTGGCATTCCGGTACTCAGCGCCCCGAGAGAGCAGC

Table S2. Lists of the GPCR sequences, their GenBank accession numbers and species used in amino acid sequence alignment.

	Chicken	Human	Pig	Giant panda	Duck	Zebrafish	Spotted gars
GPR3	-	NP_005272.1	MW310580	XP_034503372.1	MW310577	MW310585	XP_015204111.1
GPR6	MW310573	NP_001273028.1	MW310579	XP_002925693.2	MW310576	MW310584	XP_006626355.1
GPR12	MW310572	NP_005279.1	MW310578	XP_002924260.1	MW310575	MW310583	XP_015196671.1
GPR12L	MW310571	-	-	-	MW310574	MW310581<br>MW310582	XP_015206592.1

Table S3. Lists of genes and their GenBank accession numbers used to generate the phylogenetic tree in this study.

Gene names	Species	GenBank accession numbers
GPR3	Human	NP_005272.1
	Duck	MW310577
	Spotted gar	XP_015204111.1
	panda	XP_034503372.1
	pig	MW310580
	Zebrafish	MW310585
	Mouse	NP_032180.1
	Turtle	XP_006115571.1
	Finch	XP_030146224.1
GPR6	Human	NP_001273028.1
	Chicken	MW310573
	Duck	MW310576
	Spotted gar	XP_006626355.1
	Zebrafish	MW310584
	panda	XP_002925693.2
	pig	MW310579
	Human	NP_005279.1
	Xenopus	XP_017949566.1
	Turtle	XP_006123311.1
	Finch	XP_030123592.2
GPR12	Human	NP_005279.1
	Chicken	MW310572
	Duck	MW310575
	Spotted gar	XP_015196671.1
	Zebrafish	MW310583
	panda	XP_002924260.1
	pig	MW310578
	Mouse	NP_001010941.1
	Finch	XP_030130857.1
GPR12L	Chicken	MW310571
	Duck	MW310574
	Spotted gar	XP_015206592.1
	Zebrafish (GPR12La)	MW310581
	Zebrafish (GPR12Lb)	MW310582
	Japanese quail	XP_015716158.1
	Lizard	XP_028571577.1
MC4R	Human	NP_005903.2
	Mouse	NP_058673.2
	Duck	XP_005016300.1
	Chicken	NP_001026685.1

	Xenopus	XP_004915370.1
	Turtle	XP_006114320.1
	Zebrafish	NP_775385.1
	Finch	XP_030121249.1
	Spotted gar	XP_015210118.1
CNR1	Human	NP_001153698.1
	Mouse	NP_001341949.1
	Duck	XP_038033028.1
	Chicken	NP_001033741.1
	Turtle	XP_006114320.1
	Zebrafish	NP_775385.1
	Finch	XP_030121249.1
	Spotted gar	XP_015210118.1
LPAR1	Human	NP_001338326.1
	Mouse	NP_001277415.1
	Chicken	NP_001108554.1
	Duck	XP_027302018.1
	Xenopus	NP_001096313.1
	Turtle	XP_006118052.1
	Zebrafish	NP_001004502.2
	Finch	XP_002189340.1
	Spotted gar	XP_006626630.1
GHRHR	Human	NP_000814.2
	Pig	NP_999200.1
D1A	Human	NP_000785.1
	Mouse	NP_001278730.1
D1B	Human	NP_000789.1
	Rat	NP_036900.1

Table S4. Amino acid sequence identity of GPR3, GPR6, GPR12, GPR12L among vertebrate species including chickens, humans, ducks, zebrafish, and spotted gars.

		GPR3						GPR6							GPR12							GPR12L				
		human	pig	giant panda	duck	zebrafish	spotted gar	chicken	human	pig	giant panda	duck	zebrafish	spotted gar	chicken	human	pig	giant panda	duck	zebrafish	spotted gar	chicken	duck	zebrafish (GPR12La)	zebrafish (GPR12Lb)	spotted gar
GPR3	human	100.00%																								
	pig	95.10%	100.00%																							
	giant panda	96.60%	94.50%	100.00%																						
	duck	65.70%	66.30%	65.40%	100.00%																					
	zebrafish	45.20%	44.90%	45.50%	49.60%	100.00%																				
	spotted gar	63.90%	64.50%	65.10%	74.00%	50.90%	100.00%																			
GPR6	chicken	55.90%	56.20%	57.40%	61.40%	45.90%	67.40%	100.00%																		
	human	52.10%	52.10%	52.40%	56.30%	41.80%	57.70%	67.10%	100.00%																	
	pig	50.50%	50.20%	50.80%	54.60%	42.70%	56.00%	67.00%	93.90%	100.00%																
	giant panda	51.60%	51.60%	51.90%	55.20%	43.30%	57.10%	67.90%	94.40%	94.50%	100.00%															
	duck	56.50%	56.80%	57.70%	61.70%	45.60%	66.50%	96.30%	67.10%	66.70%	67.40%	100.00%														
	zebrafish	53.40%	53.10%	53.70%	59.10%	44.50%	65.40%	74.80%	61.60%	60.70%	62.90%	74.20%	100.00%													
	spotted gar	54.00%	53.70%	54.30%	59.20%	43.20%	64.90%	79.80%	62.10%	61.80%	63.20%	79.50%	81.60%	100.00%												
GPR12	chicken	55.50%	56.10%	55.80%	62.10%	48.60%	65.30%	61.70%	51.10%	51.00%	51.60%	62.00%	59.50%	57.90%	100.00%											
	human	54.10%	54.70%	54.70%	62.10%	48.00%	65.30%	62.60%	52.20%	51.60%	53.00%	62.00%	60.40%	58.20%	88.30%	100.00%										
	pig	53.80%	53.80%	55.00%	61.20%	48.30%	65.00%	62.60%	52.20%	51.90%	53.30%	61.70%	60.70%	59.10%	87.10%	95.80%	100.00%									
	giant panda	53.80%	54.40%	55.00%	62.10%	48.00%	65.60%	62.30%	51.90%	51.60%	52.70%	61.70%	60.70%	58.50%	88.90%	97.90%	97.30%	100.00%								
	duck	55.50%	55.80%	56.40%	61.80%	47.70%	65.60%	61.40%	51.10%	51.00%	51.60%	61.40%	59.80%	57.90%	94.90%	91.60%	90.40%	92.20%	100.00%							
	zebrafish	54.80%	55.30%	54.50%	60.40%	45.90%	62.70%	59.80%	51.60%	51.30%	52.10%	59.80%	55.80%	56.80%	74.40%	73.30%	71.80%	73.00%	74.10%	100.00%						
	spotted gar	53.80%	54.10%	54.70%	59.70%	47.90%	64.40%	60.50%	51.30%	50.80%	51.90%	60.50%	59.80%	58.10%	85.70%	85.10%	85.10%	86.90%	86.60%	78.00%	100.00%					
GPR12L	chicken	45.10%	45.10%	46.00%	48.20%	43.30%	50.20%	48.40%	42.70%	42.20%	42.40%	47.30%	47.80%	45.70%	54.00%	53.50%	53.50%	53.70%	54.00%	50.90%	52.60%	100.00%				
	duck	43.70%	43.70%	44.20%	46.90%	40.80%	47.20%	46.10%	42.00%	41.10%	41.80%	45.00%	45.40%	44.00%	51.70%	51.20%	51.20%	51.20%	51.40%	49.20%	50.60%	84.00%	100.00%			
	zebrafish (GPR12La)	47.30%	47.00%	48.20%	51.10%	42.80%	52.90%	51.10%	41.60%	41.10%	42.20%	50.80%	49.40%	47.40%	54.00%	54.00%	54.00%	54.30%	54.30%	50.20%	53.10%	54.50%	52.50%	100.00%		
	zebrafish (GPR12Lb)	47.90%	47.90%	49.40%	51.60%	44.10%	56.90%	53.30%	43.70%	43.40%	44.20%	52.20%	52.00%	51.40%	59.50%	58.40%	57.80%	58.40%	58.70%	54.90%	56.90%	61.60%	58.40%	66.50%	100.00%	
	spotted gar	48.30%	48.60%	48.90%	53.80%	44.10%	56.50%	54.10%	43.10%	42.90%	43.40%	53.50%	53.60%	53.70%	61.20%	60.30%	59.10%	60.30%	60.30%	55.60%	58.60%	63.90%	60.80%	66.70%	85.40%	100.00%

−3050	TCCACCAAGGTACAAGCTTCAACATTTAAATCATCATATACATATGACATAGGAGTATAGACCAGTTCCTGTCAGAGAAGTTGGTCATCTCAACTATTATAACTG
−2945	AACACACCTTTCAGAAACATTCTCAGAAATATTCCCCATGCTTTTAAGACTCAGGATGCCCATTATCTCATTTTGAATACCTAGGATTATCAACGCGCCTTGAAT
−2840	CATTTTCAGCTTTGAGATATTACTCTGCCAGCGAACAGAGCAACCAAATACAGCTGGAAGCCAGCCCTTCCACCTATGCATGTGTAATATAATGATTCATAGTAC
−2735	AAACTTTTGCCATAAGAACACACACCTTTTAGTATTTTTATTATGTATGTCTTGTTTTCTGGCTTAAAAAGCCTGAGAAGTGAGCACAGAACCAATGCCCTCTGT
−2630	GGTCCTATGATAGGACTAGAGGGAATGGCTTCAAGCTGCAGCAGGGGAGGTTCAGGCTGGACATTAGGAAATACTACTCTGAAAGAGTGGTCAGGCACTGGAATG
−2525	GGCTGCCCGGAGAGGTGTGGAGTCACCGACCCTGGTGGTGTTCAAGGAACATTTGGACATTGTGTTGAGGGACATGGTTTAGTGAGAACCATTGGTGATGGGTGG
−2420	GTGGTTGGACTCGGTGATCCTGTAGGTGTTTTCCAACCTTGGTGAATCTGTGATTCTATATTTTGTGGTGGCCTGAAGAAGCTCACTGCATATCACGTTCATCTC
−2315	AAGCTTTATTCACAATTAGTAATCAGAGACTGGCATGAAAGCACTTTTTGTAGATGTAACAAAAAATAGATGAAAGATGTACAGAAGCATAAACAGTGTGCACTG
−2210	TACCCAGTTCTAGAATATTCCAGCCATGACAAATAAAAAAAGGGGGGGAGGGGGGGGAGCAAAATAGTTATTTAATGTACACATGCAGAAGAAGTAAATCACTGT
−2105	GCTTTGTTAAAGGAAGCAGGAAAGAGAAACAGTGTTTGGGGAAAAAAAAAAAGCAAAGCAGATATACATGCAGGGAAACATGTCCCCAAGTCAGGTACTTTTGAA
−2000	GATTCCAAACACTGAATCAATTATCAGATAAAATAGAGTCCTCTGACAGAGGATGCCTCATATTAGACATGGCTATTGAGAGCAGTTGGCAACCAAGTGAGAAGG
−1895	AATGGCTTTCAAACACCTAAGGCCATTGTGTTCTTTTACCTGAGGAGAATAAACTGTGAGTTATGAGAAAATTTTCCCAGCTATCATAGCCTATAAAGAAATAAA
−1790	AATCAACTGCTACTAATTTGGGGTACATTTTTTATTCTGGCTTTTGCCCATGTAGAACTTTTATTTCATAAAGACTTCTTCCTAAAAGGTATTAAGAGGAAATCA
−1685	TTTGTGCTGGTTGTACTGTTCCAGTGATTAATTATCTTGCTATCGAATGTGCTTGATTTTGAATCTGAACTTCTCTAACTTTGGCTTCCAGGCATTATACCCTTT
−1580	TCTGTTAGAGGCATATAAAATCCTGGTGAAAGAATGCTCTAGTCCAGTCATCTCTCAGTTTTCTTCTAGAGAAACTGAAAAGAGTACTTGTATAAATCCTTCACT
−1475	ATGAGGTAATTTCTCAGGTCTTCAATGCCATATCTGTAACTTTTCTCTGAATTCTCTCTTCTTTCTTTCTCAGAACATTAAAAAAAAAAAAAATATGTATCTAGCT
−1370	TTCAAATACTAATCTCATCAGTGCCCTGTCCAGAGGTAACATAGAATCATAGGGCTTGGAAGGAAACTCTGGAGATCATCCAGTACAACCTCTGCTAAAGCAGGT
−1265	TCCCTGCTAACACAGGTGGAGCAGGAAAGCACACAGGCAGGTCTTGAATATTGCCAGAGAGGGAGGCTCCACCACCTCTCTGGGTCACCTGTTCCAGTGCTCTGT
−1160	CACCCTCAAAGTAGAGAAGTTCTTTTGCATGTTTGAATGTAACTTTTTGCATCCCAGTTTTTGCTCATCATCCCTTGTTCTGTCACTGTATACCACTGACAGGAG
−1055	CCTGGCTTCAACCATTTGACTCTCACACCTCAGGTATTTATGAACAACGAAGAGATCCCCTCTGTCTTCTTTTCTCCAGACTCAACAGCTCCAACTCTCTCAGCC
−950	TTTTCCCATAAGGAAGGTGCTCCAGGCCCCAGTCGTCTTCGTGGTCCTCAGCCCAACTCTCTCCAATAGTTCCCTGTCTTCCTTGAACTGAGGAGCCCAGAACTA
−845	GACACAGCACTCCAGATGTGGCCTCATTGGGCCCGAGTAGAGGGGGAGGATCACCTCTCCGGACCTCCTGGCCACACTCTTTGTAATGTACCCCAGGATCCCACT
−740	GGCATTCTTGACTACAGGGGCACACTGCTGGCTCATGGCCAACCTGCTGCCCAACGGAACACCCAGGTCCTTCTCTAAAGAGCTCCTTGTCAGCAGGTCAGCCCC
−635	GAGCCTGTACTGGTGCATGTGGTTATTCCTCTCCATTTGCAGAACTCTACACTTGCCCTTGTTGCCCTTTTCCTCTCTACCCAACTCTCCAGTATGTCAGGTTCC
−530	TGCTGAATGGCAGCACAGCTTTCTAGCTTCGTATCATCAGCAAACCTGCTGTGGGTGAACTCTACTCTTTTGTCCACGTCACTTTTGAATATGTTGAACAAGACC
−425	AGATCTGGTACTGACCTCAGGGAAACACCACTAGTTATAGCTCTCCAACTAGACTGTGTGCTGCTGACCACAACCCTCTGAGCTCTGCCACTCAGTCAGTTCTCA
	**CAAT**
−320	GTCC**ACCTCACTGCTTACCCCACACCCCC**AACATCACATCCTTGTTCCTGTTCGCGGCTCTCCCTATATCACAGAGCCCTCTTTGTTACAGCCCTGCACAAGGAG
	**CAAT**
−215	CTCTCGTATATAGCCGACCGGGC**ACTGAACTCCCACCCGCTCCCTCAG**CGGCGCCCTCCCGCCTCGCAGCAGGAGGCATCTGCGCAGGCGCGCGAGCACGAGGCG
	**CAP** **E2F**
−110	AACCCCCGTTCCCGCGCAGAGTCCACGTGGCCGCGCGCGCGCCGCCTACCCCTCTCCTCTCCTCTTC**TCACCCGA**ACCG**CTGCGCGC**GCAGGGGAAGGGGAGGGG
	+1 **SP1**
−5	GGGGC**G**AGCGCGCGCACCTGCGCTGCGCCCTC**GGCCCCTCACGCC**GAGCGGTGGAGGACGGCCGCCTCGGAGGGACCCTGCGAGCCTCGTCTCCCGTCCTCCGGG
	**SUF** **AP2** **SP1**
+101	AGAGC**CCAGGTGT**GTGCGTGCCGGGGGGGGGCTGCCCGGGGGTTGCGGT**GCCCGCGCGGAG**TTTGTGAGGACCTCGGAGGACCTCGCGCC**CCCCTCCCCC**ACCCT
	**AP2** **SP1**
+206	CCCT**CCGGGCGGGGCT**TTCCTTACAGGGGGTCGGGGAAGG**ACCGCCGCCCCCT**CACCCGTAACGCTCACCGC

Supplemental Fig. S1 Analysis of the promoter region of cGPR12. The nucleotide sequences of cGPR12 promoter region. The predicted binding sites for transcriptional factors were shaded. The transcription start site 'G' identified by 5'-RACE was boxed and designated as '+1'.

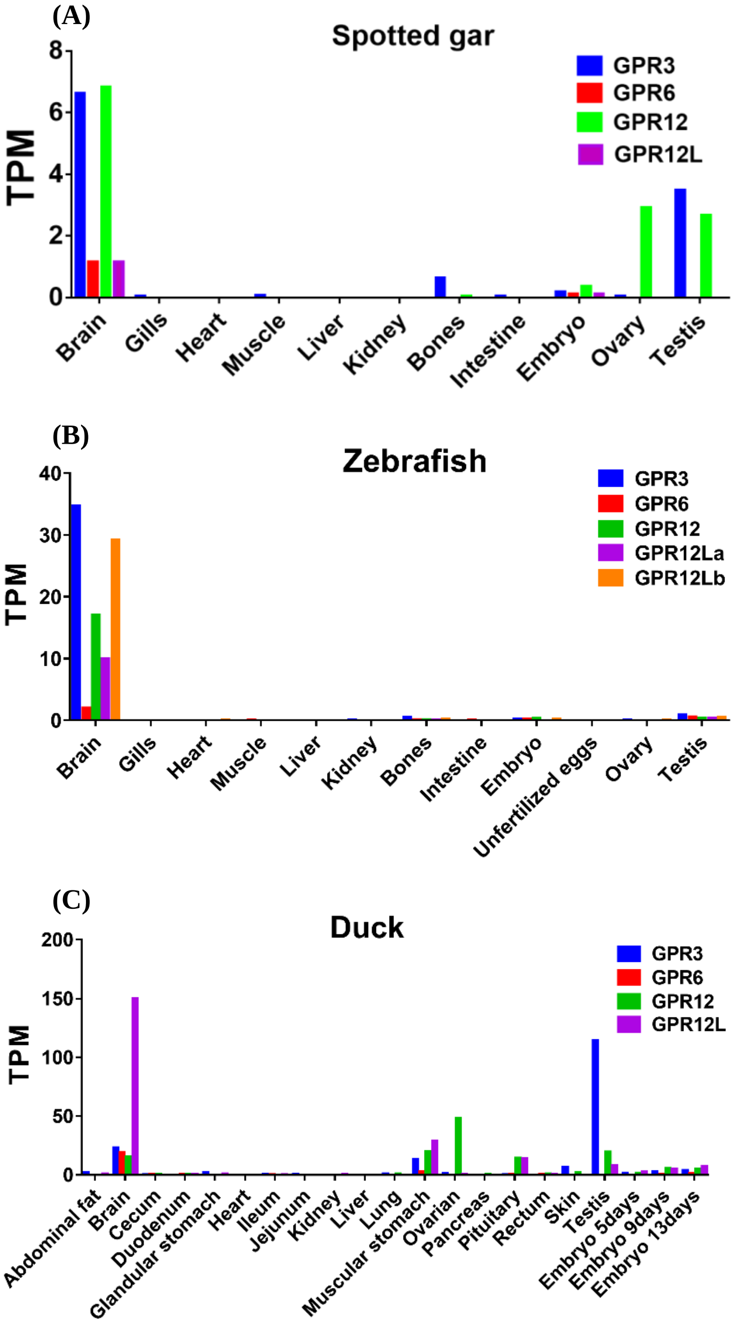

Supplemental Fig. S2 RNA-seq data analysis showing the tissue expression of *GPR3, GPR6*, *GPR12*, *GPR12La (GPR185a), and GPR12Lb (GPR185b)* in spotted gars (**A**), zebrafish (**B**), and duck (**C**) tissues.
